# Therapie der isolierten Sprechapraxie mit repetitiver transkranieller Magnetstimulation (rTMS)

**DOI:** 10.1007/s00115-022-01261-x

**Published:** 2022-03-10

**Authors:** Svetlana Politz, Ludwig Schelosky

**Affiliations:** 1 Klinik für Neurologie und klinische Neurophysiologie, Kantonsspital Münsterlingen, Spitalcampus 1, 8596 Münsterlingen, Schweiz; 2 Universität Zürich, Zürich, Schweiz

## Hintergrund

Eine isolierte Sprechapraxie ist eine erworbene neurogene artikulatorische Störung, bei welcher die sprechmotorische Programmierung geschädigt ist. Die Ursache der Sprechapraxie liegt meist in einer Perfusionsstörung im Versorgungsgebiet der linken Arteria cerebri media, insbesondere im Bereich des Gesichtsareals innerhalb des Motorkortex, des Operkulums und der Inselrinde einschließlich des assoziierten Marklagers. Eine isolierte Sprechapraxie zeigt sich klinisch nur bei 10 % der Patienten nach einem Schlaganfall.

Die Sprechapraxie äußert sich durch Suchverhalten, Sprechanstrengung und Fehlversuche mit inkonstanten und inkonsistenten phonematischen und phonologischen Fehlern. Es liegt eine Störung in der Programmierung und Koordination der erforderlichen Mundbewegungen vor [8, 10].

Im Gegensatz dazu steht die Dysarthrie, die eine durchgehende Veränderung des orofazialen und oropharyngealen Muskeltonus aufweist. Ebenfalls ist die Störung von der Aphasie abzugrenzen, gehört sie vielmehr zu den ideomotorischen Apraxien und bezeichnet eine gestörte Umsetzung komplexer Bewegungsabläufe. Bei der Apraxie kann sich der Betroffene bestimmte Bewegungsabläufe nicht mehr vorstellen, wodurch es sekundär zu einer Störung der Bewegungsplanung kommt [1].

Die rTMS (repetitive transkranielle Magnetstimulation) ist eine nichtinvasive Therapiemethode neurologischer und psychiatrischer Erkrankungen. Mit der Spule der rTMS werden an der Oberfläche des Kopfes gepulste Magnetfelder um 1 T (zum Vergleich: Erdmagnetfeld ca. 30 µT) ausgelöst, die im Kortex einen elektrischen Stromfluss induzieren und Aktionspotenziale in den unter der Spule liegenden Neuronenverbänden auslösen. Je nach Frequenz der repetitiven Stimulation können umschriebene Areale gehemmt oder erregt werden. Der Vorteil der navigierten Stimulation ist die punktgenaue, mit dem MRI-Bild des Patienten abgeglichene Stimulation der betroffenen Areale [6].

Die Methode ist etabliert und wird bei Depression, chronischen Schmerzen und chronischen Folgen nach Hirninfarkt eingesetzt (Level A [4]). Die Verträglichkeit ist üblicherweise gut. Nebenwirkungen wie Kopfschmerzen oder reversible Exantheme sind selten [7].

## Falldarstellung

**Figure 1:**
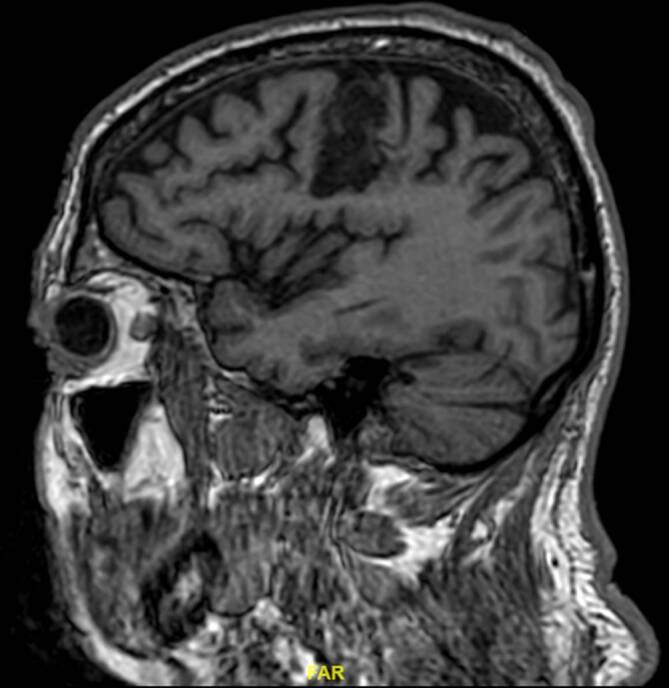

Ein 70-jähriger Mann erlitt durch eine vorher unbekannte Makroangiopathie bei hohem kardiovaskulärem Risikoprofil (arterieller Hypertonus, Hypercholesterinämie, Nikotinabusus, Adipositas) einen Verschluss der linken Arteria carotis interna mit einem ischämischen Infarkt im linken Gyrus praecentralis (Abb. 1).

Bei der Übernahme auf die Stroke-Unit bestand eine rechtsseitige brachiofaziale Hemiparese, eine globale Aphasie sowie Dysphagie. Der Patient war mit einem NIHSS (National Institute of Health Stroke Scale) von 14 Punkten schwer betroffen.

Wegen persistierender und ausgeprägter Dysphagie erfolgte die Ernährung in den ersten 5 Tagen über eine nasogastrale Sonde. Danach konnte der Kostaufbau im Rahmen des Dysphagiemanagements schrittweise umgesetzt werden.

Die kardiovaskulären, zur Makroangiopathie führenden Risikofaktoren wurden mit Amlodipin, Candesartan, Metformin und Atorvastatin behandelt. Zur Sekundärprophylaxe weiterer ischämischer Infarkte erhielt der Patient Aspirin cardio.

Nach 15-tägiger Hospitalisation auf der Stroke-Unit konnte der Patient in die 9‑wöchige Neurorehabilitation verlegt werden. Der Patient erholte sich gut. Bei der 3‑Monats-Nachkontrolle in der Schlaganfallsprechstunde zeigte sich eine noch leicht vorhandene Hemiparese rechts. Der Patient konnte sich ohne Hilfsmittel fortbewegen und Treppen steigen und zeigte einen NIHSS von 2. Wegen der Mundastschwäche rechts sowie dem Verdacht auf eine bukkofaziale Apraxie und eine Sprach- und Sprechstörung wurde die Diagnostik in der Logopädie veranlasst.

In der logopädischen Befunderhebung wurde eine bukkofaziale Apraxie mittels des Bewegungsanalysebogens nach Hartje und Poeck [2] ausgeschlossen. Die Diagnostik mittels AAT zeigte sich ebenfalls unauffällig, eine Aphasie lag nicht vor [11]. Atem- und Stimmgebung waren ebenfalls ohne Befund. Der Verdacht auf eine isolierte Sprechapraxie dagegen wurde anhand der 10-Punkte-Checkliste nach Liepold et al. bestätigt [5]. Zur qualitativen Erfassung der Symptomatik wurde eine Videoaufzeichnung angefertigt und analysiert (Video 1 und 2, siehe elektronisches Zusatzmaterial).

Die Videoanalyse zeigte Störungen auf segmentaler und suprasegmentaler Ebene sowie im Sprechverhalten. Auch bot sich das typische sprechapraktische Bild mit Inseln störungsfreien Sprechens [12].

Die Beurteilung der Verständlichkeit wurde nach Lauer und Birner-Janusch vorgenommen (0 = „vollkommen unverständlich“ bis 5 = „gut verständlich“ [3]). Vier unterschiedliche Beobachter (zwei ausgebildete Logopädinnen, eine angehende Logopädiestudentin und eine Logopädin im letzten Studienjahr) bewerteten die Verständlichkeit übereinstimmend mit dem Faktor 1 bis 2. In der gelenkten Spontansprache wurde die Verständlichkeit des Gesagten unter 2 eingestuft, während die freie Rede in der Verständlichkeit unter 1 eingestuft wurde. Beim Nachsprechen auf Satzebene wurde die Verständlichkeit mit dem Faktor 3 bis 4 beurteilt. Der Patient ordnete seine Sprach- und Sprechfähigkeit auf einer subjektiven Skala von 1 (sehr schlecht) bis 10 (unauffällig) bei 3 ein.

## Methodik

**Figure 2:**
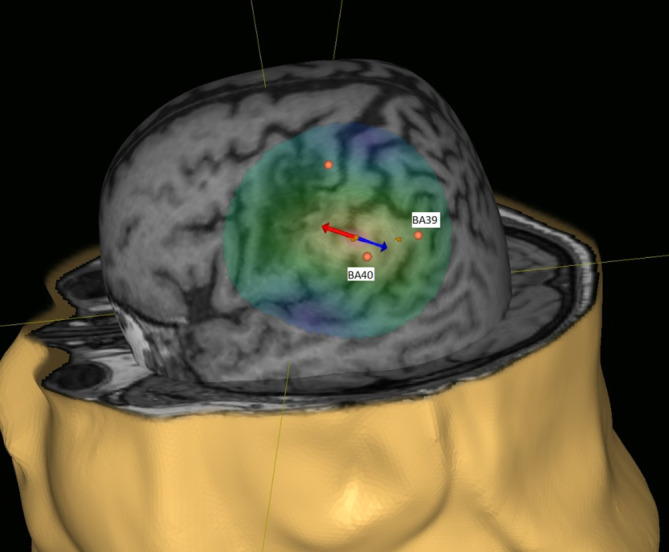

Zur Behandlung der Sprechapraxie wurde eine neuronavigierte Stimulation mit dem Nexstim Navigated Brain Therapy System NX94018‑D NBT 2.2 (Nexstim Plc., Helsinki, Finnland) durchgeführt. Das Zielgebiet lag im linken Gyrus supramarginalis (BA 40) und dem Gyrus angularis (BA 39; Abb. 2). Dort liegt das Assoziationsareal, welches eine entscheidende Rolle in der Vernetzung der Seh- und Hörzentren mit tertiären sensorischen und motorischen Assoziationsarealen spielt.

Die Stimulation erfolgte mittels einer 8‑Spulen-Geometrie in biphasischer Pulsform, beginnend am linken Gyrus supramarginalis exzitatorisch für jeweils 3′18″ mit 10 Hz, Intensität 90 % der motorischen Schwelle (MT), 1000 Pulse, 50 Pulse pro Burst, 20 Bursts pro Train, Zeit zwischen Trains 5 s. Am Gyrus angularis erfolgte die exzitatorische Stimulation über 6′36″ mit 10 Hz, Intensität 90 % der MT, 2000 Pulse, 50 Pulse pro Burst, 40 Bursts pro Train, Zeit zwischen Trains 5 s unmittelbar nach der Stimulation des linken Gyrus supramarginalis an 15 aufeinanderfolgenden Tagen (von Montag bis Freitag) in Kombination mit anschließender 30-minütiger logopädischer Therapie.

## Ergebnis

Die alleinige logopädische Therapie besserte die sprachlichen Defizite nicht ausreichend. Nach der nichtinvasiven Stimulation in Kombination mit der logopädischen Therapie konnte nach 3 Wochen eine deutliche Besserung des Sprachflusses beobachtet werden. Dies ließ sich videographisch dokumentieren (Video 1 und 2 vor, Video 3 und 4 nach Therapie, siehe elektronisches Zusatzmaterial).

Die vier vorgenannten Beobachterinnen beurteilten die Verständlichkeit nach der Behandlung übereinstimmend mit dem Faktor 4 bis 5. In der gelenkten Spontansprache wurde die Verständlichkeit des Gesagten bei 5 eingestuft, während die freie Rede in der Verständlichkeit als „weniger verständlich“ unter 4 eingestuft wurde. Beim Nachsprechen auf Satzebene wurde die Verständlichkeit mit dem Faktor 5 eingestuft. Der Patient ordnete seine Sprach- und Sprechfähigkeit auf einer subjektiven Skala von 1 (sehr schlecht) bis 10 (unauffällig) nun bei 7 ein.

Im Alltag profitierten der Patient und seine Ehefrau mit einer verbesserten Kommunikation untereinander. Darüber hinaus beschrieb der Patient eine Verbesserung seiner verbalen Interaktion mit dem sozialen Umfeld, was sich positiv auf seine Teilhabe auswirkte.

Relevante Nebenwirkungen wurden nicht beobachtet.

## Fazit

Für die Behandlung der Sprechapraxie liegt im deutschsprachigen Raum die S1-Leitlinie vor, in welcher die Sprechapraxie nur als Stichwort in „Rehabilitation aphasischer Störungen nach Schlaganfall“ Erwähnung findet [12]. Die Evidenzlage für einzelne Therapieansätze ist bisher überwiegend gering, aber es liegen Empfehlungen für das therapeutische Vorgehen aus den US-amerikanischen Leitlinien [9] vor.

Die Kombination von rTMs und Logopädie erwies sich bei unserem Patienten als wirksame Methode, seine sprachliche Ausdrucksmöglichkeit und somit auch seine soziale Teilhabe zu verbessern.

## Supplementary Information







